# Optimization of Pesticides Spray on Crops in Agriculture using Machine Learning

**DOI:** 10.1155/2022/9408535

**Published:** 2022-09-05

**Authors:** Indu, Anurag Singh Baghel, Arpit Bhardwaj, Wubshet Ibrahim

**Affiliations:** ^1^Department of Computer Science and Engineering, USICT, Gautam Buddha University, Greater Noida, India; ^2^Department of Computer Science and Engineering, BML Munjal University, Gurugram, India; ^3^Department of Mathematics, Ambo University, Ambo, Ethiopia

## Abstract

Pesticides are chemicals used to eradicate pests. Not only are they used for plant protection and livestock in agriculture, but they are also used in public areas to kill mosquitoes, cockroaches, and other pests. Approximately 95% of the pesticides produced are only used in agriculture for crop protection. Every country wants to increase crop production. To protect their crops from pests, farmers must use pesticides. Exposure to pesticides is increasing day by day, whether occupationally or environmentally. This has resulted in an increase in crop production, but it has numerous adverse effects on human health, animal health, and the environment. Farmers repeatedly use the same pesticides on their crops, which is detrimental to human health and the environment. In this research, according to authors, the repetition of pesticides in agriculture is controlled using adjuvant and machine learning algorithms. An adjuvant is a chemical agent that is inserted within the pesticide product for enhanced pesticide performance. By utilizing an algorithm for machine learning, it is no longer necessary to repeatedly spray the same pesticide over the entire crop field in order to determine which sections of the crop field still require repeated pesticide spraying. In this research, the authors predict that 72.5% of insecticides are used in India. Logical regression classification, polynomial regression, and K-nearest neighbor algorithm (KNN) are applied to detect this required field.

## 1. Introduction

According to the Environmental Protection Agency, a pesticide can be any substance that is used to kill insects, pests, fungi, and weeds, whereas it is used to preempt plants and livestock. Since pesticides are believed to be toxic, they are biologically active agents that have caused a physiological response. Pesticides are a specific type of toxins, which are applied to crops to control pests, weeds, and so on, despite being toxic to humans, animals, livestock, and the environment. Pesticides are effective in controlling pests and insects using these methods, but they pose a threat to humans, animals, and the environment. The authors insist that pesticide poisoning occurs when pesticides or other chemicals designed to control pests and insects affect nontarget organisms, such as wildlife, plants, bees, humans, and the environment [1, 2]. This is a global public health problem and pesticide acute poisoning accounts for 300,000 deaths worldwide every year. Few researchers have reported that the annual number of deaths worldwide exceeds 5 million. Experts broadly categorize pesticide effects as topical or systemic. Here, we will first define topical reactions. They are also known as topical allergy, and they are only harmful to those who have come into direct contact with pesticides. They affect a limited area of the body and their effects manifest in various ways, such as rashes, itching, blisters, scaly skin, or other forms of skin inflammation. Some major symptoms of topical reactions on humans are sneezing, wheezing, and coughing. No matter where you live, whether in a village, town, or city, or how cautious you are, there is a good chance that you have been exposed to pesticides for a long time. The beautiful, colorful vegetables and fruits that you see in the markets are grown by the use of pesticide sprays, whether you like it or not. Most people are unaware of what pesticides are and how they affect human health, despite the fact that they are extremely prevalent and can never be completely avoided. Pesticides are difficult to diagnose because their symptoms do not appear immediately. They can manifest years after initial exposure. Signs of pesticide effects on the human body include headache, fatigue, nausea, bowel problems and difficulty breathing, severe pesticide poisoning, seizures, and changes in the heartbeat.

### 1.1. Pesticides Used in Agriculture

**Figure 1:**
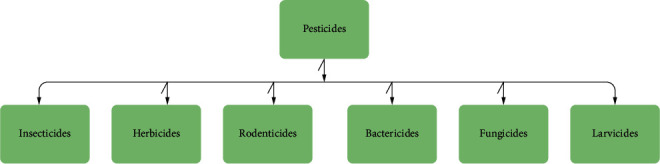
Types of pesticides.

To increase production in agriculture, pesticides play a vital role. Crops and plants are sprayed with pesticides for various reasons. Pesticides are used to protect crops and plants from insecticides [3–5]. They are also used for the growth of crops and plants. A pesticide is known as a chemical or biological agent; it deters, incapacitates, kills, or discourages pests. In India, pesticide production has been started in 1952 with the establishment of a plant BHC, which is near Calcutta. In Asia, India is the second largest manufacturer of pesticides; the first one is China. Pesticide is a term that covers a wide range of compounds; it includes insecticides, herbicides, fungicides, molluscicides, nematicides, rodenticides, and plant growth.  Figure 1 depicts many types of pesticides.

After the 1960s, the majority of technologically advanced nations halted or restricted the use of organochlorine insecticides, which were effective in combating a variety of diseases, including malaria and typhus. Some of the most well-known pesticides, including synthetic insecticides, organophosphate (OP) insecticides in the 1960s, carbamates [6] in the 1970s, and pyrethroids in the 1980s, greatly aided in the control of pests and the expansion of agricultural production. India spends a large amount on pesticides. It has spent nearly $0.6 billion annually. It is a total of 1.6% of the international pesticides market that is accessed by India, although the consumption of pesticides still has muffled in India. Widespread contamination of pesticides in food commodities, due to pesticides residue, is an indiscriminate and nonjudicious application. Notwithstanding WHO (World Health Organization) has banned the use of certain organochlorine syntheses. In many developing countries, these chemicals are intensively used in limited quantities including India. India uses these chemicals in limited quantities for agricultural, livestock, and public health programs.

### 1.2. Benefits of Pesticides in Agriculture

Agriculture is the backbone of India; it contributes to the country's progress. Populations in the majority of nations are growing exponentially, and agricultural lands are shrinking; consequently, the agriculture industry is under extreme strain. This sector has immense pressure to perform. In the past, pesticides and fertilizers came to the rescue by acting as a catalyst in stabilizing higher yields gained through the use of hybrid seeds.

This period was known as the Green Revolution, but since then it has undergone a radical transformation. Perhaps, the side effects of pesticides are considerably evident. The inclination toward moving forward is gradually diminishing. Pesticides are designed to target and eliminate insects and weeds; however, they have negative effects on living organisms and pose a threat to humans, nontarget plants, and animals. Pesticides have a negative impact on the ecosystem, as well as on food and food plants. They bioaccumulate throughout the food chain and can be detected in plant and animal tissues. They are hazardous to health. Farming, looking after animals, and food crops are one of the most important areas of foreign exchange. Indian products also can compete in international markets if they complete the scale of international standards. To increase exports, it is compulsory to establish the competitive advantages of animal product. Silage fed to animals is often contaminated with pesticide residues, which are absorbed by the body after consumption. It is required to strengthen the quality competitive aspects of animal products. According to extensive research conducted by scientists or researchers, pesticide poisoning in humans is frequently reported in forensic medicine. According to one estimated report of WHO, all over the world, 20,000 deaths are happening every year because of pesticide poisoning. One million pesticide poisoning [7, 8] cases in humans are occurring all over the world because of pesticide use. Pesticides affect human health and the environment. Pesticide residue can be detected in milk, meat, and other dairy products. Pesticides cause an increase in human health problems as well as death. They are reduced using a machine learning algorithm, as a survey field. The objective of self-sufficiency in pesticides or reduced repetition of pesticides is in agriculture eke prevention and control strategies for the occurrence of pesticides in animal products throughout the world. Short-dated pesticides poisoning or acute toxicity from pesticides is typically the result of a single poisoning that might be happening due to exposure via the skin, inhalation, eyes, or mouth; therefore, to avoid these side effects of being exposed to pesticides, it is best to avoid using such chemicals in your homes and to choose organically grown fruits and vegetables.

## 2. Problem Statement

This section described the types of pesticides as well as their side effects and then discussed methods to deduct them. Basically, there are different kinds of pesticides.

### 2.1. Organophosphate Pesticides

Organophosphate is a human-made chemical. It is an insecticide. It is used in agriculture; it is sprayed on crops and plants. Organophosphate [1, 8] is a pesticide that affects the nervous system. It disrupts the enzyme that regulates acetylcholine, a neurotransmitter.

### 2.2. Carbamate Pesticides

Carbamate pesticides are similar to organophosphate (OP). Carbamate compounds are esters of carbamic acid that are used as insecticides. Carbamate [1, 8, 9] pesticides also affect the nervous system. They disrupt the enzyme that regulates acetylcholine, a neurotransmitter. Enzymes affect reversible processes. Carbamates contain various subgroups like aldicarb, carbofuran, and carbaryl.

### 2.3. Organochlorine Insecticides

Organochlorine refers to a wide range of chemicals; these chemicals contain carbon and chlorine, as well as other elements. Organochlorine includes dichlorodiphenyltrichloroethane. It was used in the past. Lots of organochlorines have been discarded from the market due to their effect. It affects human health. It is persistent in the environment. DDT, chlordane, aldrin, dieldrin, heptachlor all are generic OC compounds.

### 2.4. Pyrethroid Pesticides

Pyrethroids are chemicals that were created to kill insects and mosquitoes. They are a mock translation of the naturally occurring periodically or repeatedly pesticides pyrethrin [1, 3, 7] scilicet in chrysanthemums. They have been modified to increase their stability in the environment. Umpteenth mock pyrethroids are toxic to the nervous system. Commonly used pyrethroids are deltamethrin, cypermethrin, and permethrin.

Tables 1 and 2 explain different types of chemical pesticides.

### 2.5. Impacts on Humans

**Figure 2:**
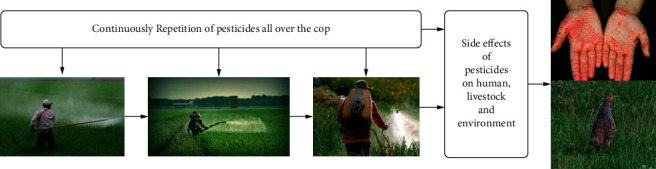
Repeated pesticide application has an impact on people, pets, and animals.

Agriculture production has been increasing due to pesticides. Exposure to pesticides is rapidly increasing. It sounds good for pesticides organization, but pesticide exposure causes numerous health problems in humans and animals. After continuously observing the side effects of pesticide exposure on the environment and the human population, it was discovered that pesticides influence immune suppression, reproductive abnormalities [10, 11], diminished intelligence, cancer, hormone disruption, and air quality as shown in Figure 2. Now there is irrefutable evidence that few chemicals pose a risk to humans and the environment. Currently, there is no population segment in the world that is completely protected from the side effects of pesticides. Still, humans are not standing against pesticides exposure [12–14]. The population of developing and under-developing countries bears a disproportionate share of the health risks, despite the fact that they may be severe. Currently, this issue is categorized as a high-risk group in every nation. It is estimated that over 20,000 people die annually due to direct contact with pesticides around the globe. Pesticides are being used in agricultural regions [11, 15]. They enter the human body after being released into the environment from agricultural land. Pesticides can have a direct or indirect effect on human and animal life in modern times. Pesticides are currently ingested, inhaled, or come into contact with the skin via foods, water, soil, and the environment. According to research, acute and chronic health problems are increasing due to exposure to pesticides [16, 17]. Few people use pesticides to get suicide. If suicides are counted alongside accidental deaths, the annual global death toll attributable to pesticide exposure exceeds 1 million. Some spray operators and farmers do not wear spraying clothes or protective clothing and equipment when applying pesticides. They use pesticides carelessly and wear inadequate clothes [18, 19]; these cases are categorized as accidental exposure. In India, the first documented case of pesticide poisoning occurred in Kerala in the year 1958 [19, 20]. According to this report, more than 100 people died after consuming wheat flour contaminated with parathion. One of the most dangerous reports came from Bhopal in 1984, where the chemical methyl isocyanate (MIC) leaked from the Union Carbide India Ltd. (UCIL's) pesticide factory and it turned the city of Bhopal into a colossal gas chamber. This accident resulted in more than 15,000 deaths in a single day and affected 60,000 people.

There are two major impacts of pesticides. They are as follows. (i) Undeveloped Brain: the cause of a newborn's undeveloped brain is the mother's consumption of pesticide-grown vegetables and fruit. (ii) Tumor Development: pesticides contribute to the spread of cancer in humans. Due to the use of pesticides on fruits and vegetables consumed by humans, a low level of poison is continuously accumulated in the human body. It complicates diagnosis, and some pesticides can cause seizures, changes in heart rate, and sometimes death.

### 2.6. Impacts on Nontarget Organisms

Pesticides have been detected as impurities in the soil, air, and water, as well as in the bodies of nontarget organisms in our metropolitan scenes. Pesticides produce copious amounts of synthetics; these synthetics could harm plants, crops, organisms, people, aquatic organisms, birds, and other forms of natural life. The use of pesticides in agriculture has a direct impact on the mortality of birds. Pesticides directly cause severe harm through ingestion of granules, draws, treated seeds, and direct exposure to sprays. By implication, the death of birds could result from the consumption of treated crops, contaminated water, or the consumption of contaminated prey. Natural life damage depends on the toxicity and other properties of a pesticide, the amount applied, the timing, the frequency, the climate, the vegetation structure, and the soil type, as well as the application technique. Insecticides, fungicides, rodenticides [21, 22], and the most toxic herbicides threaten untamed wildlife.

### 2.7. Impact on the Environment

Pesticides are a group of synthetic substances that are intentionally applied to the climate to cinch plant and creature irritations and safeguarding farming and modern products is additionally utilized. It is possible that pesticides have contaminated soil, grass, water, and other vegetation [23–25]. The major task of pesticides is to kill insects or weeds, but sometimes they can be toxic to other nontargeting insects like organisms, birds, fish, and earthworms. Nonetheless, pesticides are not precisely focusing on the irritation; they likewise influence nontarget plants and creatures. Rehashed application (redundancy of pesticides) starts to prompt loss of biodiversity. A few pesticides are not degradable; they continue in that frame of mind, to groundwater and surface water, and pollute the climate. Depending on the chemical properties of pesticides, they can enter living organisms, bioaccumulate in food chains, and influence the climate.

## 3. Related Work

Numerous research papers have described how the use of pesticides on crops is harmful to humans, livestock, animals, and the environment.

### 3.1. Banned Pesticides

Many countries have banned certain pesticides as a result of their adverse effects on the environment. These pesticides are banned to protect livestock, humans, and the environment. A much more cautious use of agrochemicals is required through testing, careful risk assessment, and licensing, as well as through farmer education and measures for greater ecosystem protection [2, 9].

### 3.2. Pesticide Toxicity Effects on Human

Pesticide toxicity is dependent on the family of compounds; it is greater for the older compounds. If we talk about humans, they have been responsible for acute poisonings, as well as long-term health effects such as cancer and adverse effects on reproduction [6, 19].

### 3.3. Crop Production and Disease Detection Using Machine Learning

A machine learning algorithm is used for crop condition identification, early disease detection, targeted crop protection, and recommendation. It is a way to focus on how the recommender system is used in agriculture for disease detection and prediction [5, 26].

## 4. Methodology

The methodology section contains the adjuvant and the machine learning.

### 4.1. Adjuvant

An adjuvant is a chemical agent that is inserted within the pesticide product or pesticide spray mixture for enhanced pesticide performance. An adjuvant is not used to control pests; it does not have any pesticide properties, so there is no need to register it in the US Environmental Protection Agency (EPA). One of the most effective uses of adjuvant is reducing pesticide spray.

An adjuvant can be combined with pesticides in two ways.

### 4.2. Formulation Adjuvant

Adjuvant for formulation was a component of the pesticides created by the manufacturer.

### 4.3. Spray Adjuvant

Spray adjuvant is not combined with pesticides by the manufacturer. Compared to pesticides, it is more tailored. Spray adjuvant is added in pesticides spray by the applicator. The below tables could serve as a guide for general rainfastness to complement a comprehensive pest management decision-making process. The amount of pesticides needed for each level is listed in Table 3.

### 4.4. Machine Learning

Using a machine learning algorithm, crop data after pesticide application is analyzed to determine which areas of the crop require a second application of pesticide. There is no need to reapply pesticide to the entire crop.

## 5. Implementation of Model

The implementation model includes a flow chart and a block diagram for the machine learning approach.

### 5.1. Flow Chart

**Figure 3:**
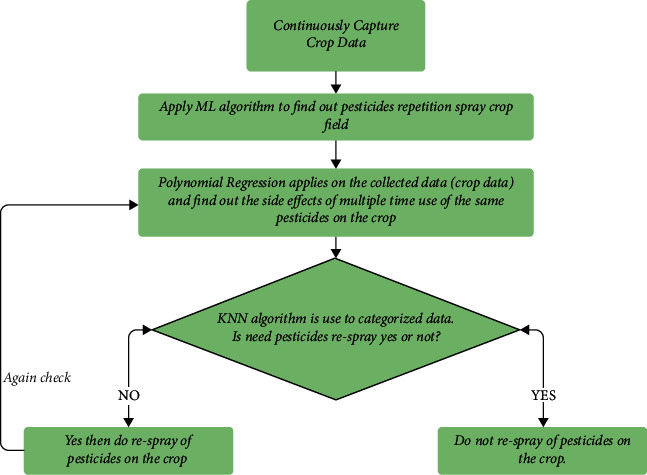
Flow chart illustrating the steps of the process to identify the necessary crop areas for repeated pesticide application.

In this section, the working process is explained step by step using a flow chart.  Figure 3 explains each step one by one.

1. Capture continuously crops data following pesticide application.
2. Captured data is processed using machine learning to find out which crop area requires a repeated application of pesticide. Using machine learning algorithms such as KNN [27–29] eliminates the need to repeatedly spray the entire crop field.
3. Do spray. And repeat steps 2.1 and 2.2.
4. If not required, stop.

### 5.2. Machine Learning Approach

**Figure 4:**
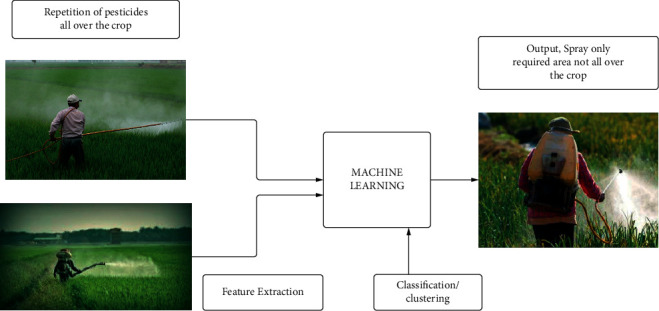
The block diagram illustrates how machine learning may be used to cut down on the use of pesticides on crops.

The inputs of machine learning are repeated crop pesticide data and previous data extracted from feature extraction. An algorithm is then applied to the input data to optimize the output, as shown in Figure 4.

## 6. Results and Discussion

In the results section, the exact amount of pesticides needed on the market is indicated.

### 6.1. Pesticides

**Figure 5:**
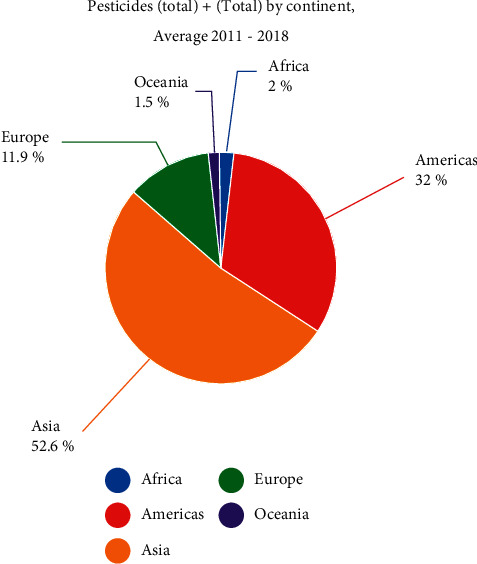
Use percentages to illustrate how widely pesticides are used. Asia is the region that uses pesticides the most.

Pesticides are chemical substances, which are utilized to kill bugs. Pesticides are used to eradicate bugs and to protect plants and animals from them. Firstly, we are examining the use of pesticides in the chart below. Compared to the rest of the world, 52.6% of pesticides are consumed in Asia, placing Asia in first place. 32% of pesticides are consumed in the Americas, placing them in second place. In Europe, 11.6% of pesticides are consumed . In Africa, 2% of pesticides are expended. In Oceania, 1.5% of pesticides are expended. Asia is in first place. The below figures show the total amount of pesticides used on each continent, whereas Table 4 shows pesticide usage by country including the domain name, domain area, unit, and value. Italy is the first of the top ten nations that use pesticides globally. Poland is the second, as seen in Figure 5. There is a slight difference between Italy and Poland, if we compare them. Brazil is in the third place and Ukraine is in the fourth place. Germany is in the fifth place. United Kingdom of Great Britain and Northern Ireland is in the sixth place. Uruguay is in the seventh place. Tajikistan is in the eighth place. Malaysia is in the ninth place. Sudan is in the tenth place.

**Figure 6:**
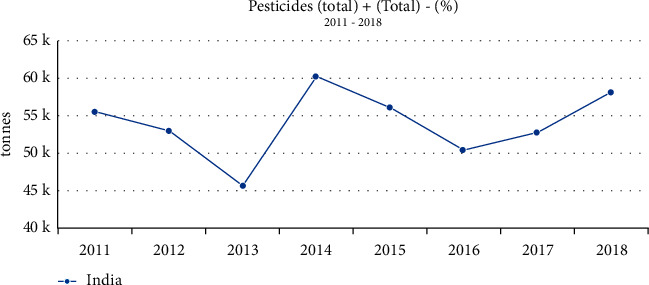
Using a graph showing the total amount of pesticides used in India from 2011 to 2018.

**Figure 7:**
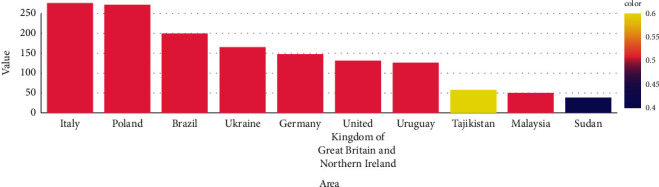
Top 10 countries worldwide with the highest pesticide use.

We are clarifying the utilization of pesticides in India, Asia. India is second to China in Asia in terms of the use of pesticides, which is enormous. The chart discusses how many tonnes of pesticides India consumes annually. In this chart, data is taken care of between 2011 and 2018 as shown in Figure 6. The use of pesticides decreased in 2014, but they are still being used today. Across the globe, ten countries are utilizing excessive amounts of pesticides as shown in Figure 7. An explanation of the pesticides used in the nation together with the domain name, code, region, and the number of units utilized along with the values attained is shown in Table 4.

### 6.2. Insecticides

**Figure 8:**
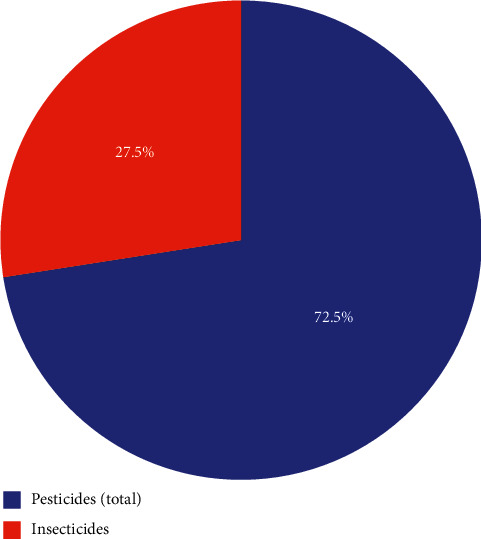
Total contribution of insecticides from pesticides in India.

Pesticides is a term that encompasses a variety of compounds; it incorporates bug sprays, herbicides, fungicides, molluscicides, nematicides, rodenticides, etc. 72.5% of the market for pesticides is comprised of pesticide sprays. Insecticides are utilizing 27.5% in agribusiness, as shown in Figure 8; it is a huge sum since bug sprays have been related to cancer. Based on item code, value, and flag, pesticides and insecticides differ from one another, as shown in Table 5. In India, bug spray use has been consistent for a long time. Table 5 shows the total use of pesticides and insecticides based on some factor such as item, item code, unit, value, and the flag. India's total insecticide contribution is from pesticides, as shown in Figure 8.

**Figure 9:**
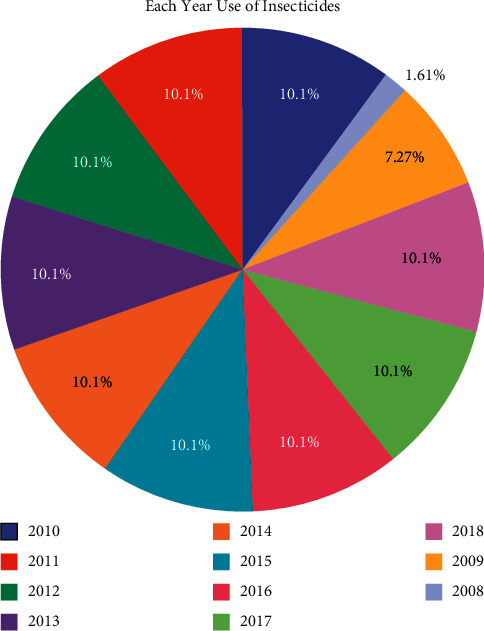
Annual use of insecticides in India.

In 2008 insecticides sprays employments were 1.61%, but, in 2009, they increased to 7.27%. Once more they have been expanded in 2010, bringing the total to 10.1 to date, as shown in Figure 9.

This section focuses on the personal use of insecticides, bactericides, herbicides, and fungicides in India. 32.6% are fungicides and bactericides, 51.5% are insecticide sprays, and 15.8% are herbicides.

### 6.3. Fungicides

**Figure 10:**
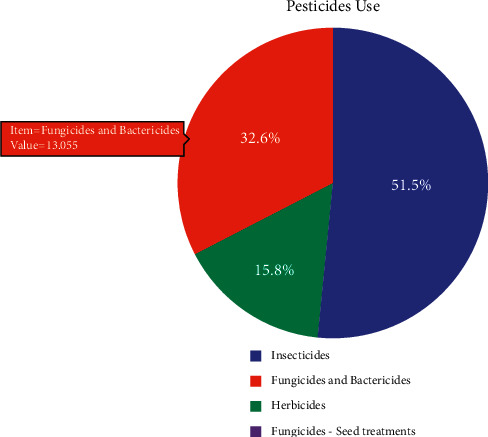
Individual contribution of insecticides, bactericides, herbicides, and fungicides in India.

**Figure 11:**
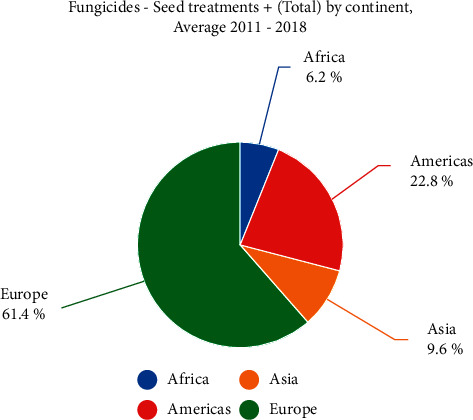
Total contribution of fungicides all over the world. Europe is on top to use it.

This study identifies the countries that use the most fungicides, based on survey data collected from 2011 to 2018. The amount of fungicides used increases annually. The most hazardous fungicide was vinclozolin, which is no longer used. Long-term human exposure to the fungicide Ziram is nephrotoxic, and ingestion is lethal. Fungicide causes skin and eye irritation, and ingesting an excessive amount of it causes nausea, vomiting, and tissue damage. Ziram does not induce cancer in humans, but its long-term exposure effects cannot be detected in an early stage. A high dose of toxic copper sulfate may endanger humans, animals, and the environment. Europe utilizes 61.4 % of the world's fungicides; with this massive quantity it is in the first place. The Americas are in second place and consume up to 22.8% of fungicides, as shown in Figure 10. Asia is in the third place (9.6% of fungicides). Africa is in the fourth place, and it consumes 6.2% of fungicides, as shown in Figure 11.

## 7. Conclusion

In agriculture, the amount of pesticides, insecticides, and fungicides sprayed annually to kill pests, insects, weeds, and fungi kills nontargeted animals, birds, and people as well. Farmers repeatedly apply pesticides to the same crops in order to protect them from pests. Certainly, farmers save their crops by doing so, but the repeated use of pesticides has a number of negative side effects, such as brain underdevelopment, cancer, seizures, heart problem, eyes problems, and lung cancer. In this research paper, the data from pesticides sprayed on crops is processed using an algorithm for machine learning, and the area that does not need pesticides sprayed again is centroided so that farmers only apply pesticides to the required area. This concept reduced pesticide repetition by at least 20% compared to the exit concept. Humans, animals, and birds must be protected from repeated pesticide use in agriculture.

## Figures and Tables

**Table 1:** Explanation of insecticides persistence, plant penetration, and rainfastness rating.

Insecticides persistence, plant penetration, and rainfastness rating				
S. No.	Insecticides group	Persistence	Penetration	Rainfastness rating
1	Organophosphate pesticides	Medium-long	Surface	Low
2	Carbamate pesticides	Short	Cuticle	Moderate
3	Organochlorine insecticides	Long	Surface	High
4	Pyrethroid pesticides	Short	Cuticle	Moderate-high

**Table 2:** Types of chemical pesticides.

Chemical pesticides						
S. No.	Name	Pesticides types	Compounds	Area	Mixed	Toxic
1	Organophosphate	Organic compounds	Household	Commercial insecticides	Water & oil	Toxicological action alters neuromuscular synapses

2	Carbamate	Mostly insecticides	Esters of carbamic acid	Kill insects by affecting their brains and nervous systems	Water highly significant (*P* < 0.05)	Toxicological action alters neuromuscular synapses in a reversible way

3	Organochlorine	Insecticides	Hydrocarbons with more than one chlorine atom	Agriculture and mosquito	Water	DDT, methoxychlor, dieldrin, chlordane, toxaphene, mirex, kepone, lindane, and benzene hexachloride

4	Pyrethroid	Insecticides	Chrysanthemum flowers	Household insecticides, pet sprays, and shampoos	Water & oil	Permethrin, resmethrin, and sumithrin

**Table 3:** General characteristics for insecticides chemical classes.

Insecticides group	Rainfastness ≤0.5 inch (1.25 cm)		Rainfastness ≤1 inch (2.75 cm)		Rainfastness ≤2 inches (4.5 cm)	
	Crop	Root	Crop	Root	Crop	Root
1	M	M/H	M	M	L	L
2	L	M	L	M	L	L
3	M/H	M/H	M	M	L	L
4	H	H	H	M	M	L

H-High rainfastness (≤30% residue wash = off), M-Moderate rainfastness (≤50% residue wash-off), L-Low rainfastness (≤70% residue wash-off), S-Systemic residues remain in plants.

**Table 4:** Explanation of pesticides used in the country with the domain name, code, area, and how many units with values are obtained.

S.No.	Domain code	Domain	Area code	Area	Element code	Element	Unit	Value	Flag
1	RP	Pesticides use	106	Italy	5157	Agricultural use	tonnes	275.63	A
2	RP	Pesticides use	173	Poland	5157	Agricultural use	tonnes	273.50	A
3	RP	Pesticides use	21	Brazil	5157	Agricultural use	tonnes	198.88	A
4	RP	Pesticides use	230	Ukraine	5157	Agricultural use	tonnes	165.13	A
5	RP	Pesticides use	79	Germany	5157	Agricultural use	tonnes	147.50	A

**Table 5:** Differences between pesticides and insecticides are based on item code, value, and the flag.

S.No.	Domain code	Domain	Area code	Area	Element	Item	Item code	Unit	Value	Flag
1	RP	Pesticides use	100	India	Agricultural use	1357	Pesticides (total)	tonnes	56120	A
2	RP	Pesticides use	100	India	Agricultural use	1357	Pesticides (total)	tonnes	50410	A
3	RP	Pesticides use	100	India	Agricultural use	1357	Pesticides (total)	tonnes	52750	A
4	RP	Pesticides use	100	India	Agricultural use	1357	Pesticides (total)	tonnes	58160	A
5	RP	Pesticides use	100	India	Agricultural use	1309	Insecticides	tonnes	20619	F

## Data Availability

The data can be obtained from the corresponding author upon request.
